# Long non-coding RNA HOTAIR promotes tumorigenesis and forecasts a poor prognosis in cholangiocarcinoma

**DOI:** 10.1038/s41598-018-29737-4

**Published:** 2018-08-15

**Authors:** Wei Qin, Pengcheng Kang, Yi Xu, Kaiming Leng, Zhenglong Li, Lining Huang, Jianjun Gao, Yunfu Cui, Xiangyu Zhong

**Affiliations:** 1 Department of Hepatopancreatobiliary Surgery, Second Affiliated Hospital of Harbin Medical University, Harbin, China; 2 The Key Laboratory of Myocardial Ischemia, Harbin Medical University, Ministry of Education, Heilongjiang Province, China

## Abstract

Cholangiocarcinoma (CCA) arising from the neoplastic transformation of cholangiocytes with increasing incidence in the worldwide. Unfortunately, a large amount of CCA patients lost their chance for surgery because it is hard to diagnose in the early stages. Long non-coding RNAs (lncRNAs) is closely associated with development and progression of various malignant tumors. Hox transcript antisense intergenic (HOTAIR), a negative prognostic factor for patients with gastric, liver and pancreatic carcinoma. Its transcription levels and functional roles in CCA is still unknown. Therefore, we aimed to explore the effect of HOTAIR in CCA including cell proliferation, apoptosis, migration, invasion and epithelial-to-mesenchymal transition (EMT). The results showed that HOTAIR was highly expressed both in CCA tissue samples and cell lines compared with corresponding normal bile duct tissues and Human intrahepatic biliary epithelial cells (HIBEC). Its overexpression was closely correlated with Tumor size, TNM stage and postoperative recurrence in CCA patients. Moreover, up-regulation of HOTAIR has correlation with prognosis in CCA patients. Knockdown of HOTAIR by siRNAs significantly decreased the migration and invasion but increased apoptosis of CCA cells *in vitro*. Overall, our study revealed that HOTAIR may play as a new potential therapeutic target and forecast poor prognosis for this fatal disease.

## Introduction

Cholangiocarcinoma (CCA) is a common malignant tumor arising from the neoplastic transformation of cholangiocytes with increasing incidence all over the world^1,2^. For patients with CCA, surgical resection remains the best therapeutic approach. However, this deadly disease is frequently accompanied with poor prognosis, even more unfortunately, most patients with CCA lost their chance for surgery because of lack of early diagnostic evidence^3^. In addition, the majority of patients are insensitive to chemotherapy and radiotherapy and the survival rate for curative-intent resection is dismal. Therefore, elucidating the mechanism of CCA, identifying the pathogenic factors and finding a new treatment target are urgently needed.

With the advance of gene technology, it has been found that long non-coding RNAs (lncRNAs) is closely associated with development and progression of malignant tumours^4,5^. LncRNAs, composed of at least 200 nucleotides and do not have the potential to encode functional proteins^6,7^. Many studies found that dysregulated expression of lncRNAs play imperative roles in cancer suppressor gene or oncogenes. Many LncRNAs have been well studied in CCA, for example, Taurine-up-regulated gene1 (TUG1), a 7.1-kb lncRNA, plays oncogenic roles in CCA^8^.

Hox transcript antisense intergenic (HOTAIR) is a 2158-bp long intervening non-coding RNAs^9,10^. Recently, HOTAIR has been determined to be overexpressed in most kinds of human malignancies and closely associated with tumor development. It has been affirmed that HOTAIR is a negative prognostic factor for gastric, liver and pancreatic carcinoma. Moreover, HOTAIR is correlated with ambitious ovarian and esophageal cancer metastasis^11–15^. Meanwhile, knockdown of HOTAIR can inhibit cell invasion and proliferation^16–20^. The findings above indicated that HOTAIR plays imperative roles in the modulation of cancer progression. Nevertheless, little is known about the impact of HOTAIR on CCA carcinogenesis or metastasis.

In order to understand the functional role of HOTAIR in CCA development and progression, in our study, we investigated the expression levels of HOTAIR in CCA tissues and analyzed its connection to clinical and pathological characteristics. In addition, we investigated the crucial roles of HOTAIR in CCA by analyzing proliferation, apoptosis, migration, invasion and epithelial-mesenchymal transition (EMT) after silencing of HOTAIR *in vitro*.

## Results

### HOTAIR is overexpressed in human CCA cell lines and tissues

**Figure 1:**
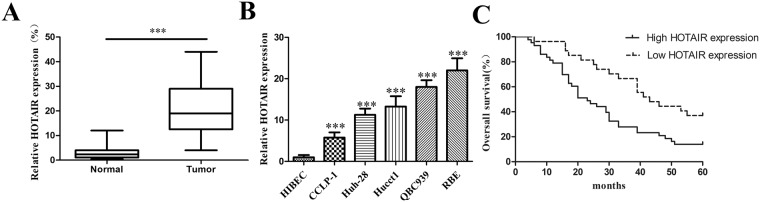
Relative HOTAIR expression in both CCA tissues and cell lines and its clinical significance in patients with CCA. (**A**) Relative HOTAIR expression levels in tumor and normal tissues; (**B**) HOTAIR expression levels in CCA cell lines and HIBEC; (**C**) Kaplan–Meier survival analysis applied based on postoperative survival time and HOTAIR expression. ^***^p < 0.001.

In order to elucidate the expression levels of HOTAIR in CCA, 70 pairs of tissues and diverse CCA cell lines including HuCCT1, CCLP-1, Huh-28, QBC939 and RBE were investigated by qRT-PCR experiments. The experimental results showed that the relative expression of HOTAIR in CCA specimens was obviously superior than that of the corresponding normal tissues (Fig. 1A). Besides, the expression of HOTAIR was up-regulated in CCA cell lines compared to HIBEC cells (Fig. 1B). The fact above indicated that HOTAIR might play key roles in tumorigenesis and progression in CCA.

### Up-regulated HOTAIR is negatively correlated with prognosis

70 patients were divided into high HOTAIR expression group or low expression group. Clinicopathological factors were analyzed between the high and low HOTAIR expression groups. As a result, the high HOTAIR expression group showed higher postoperative recurrence (*p* = 0.020), larger tumor size (*p* = 0.028) and more advanced TNM stage (*p* = 0.021) than the low HOTAIR expression group. However, patients’ gender, age, lymph node invasion and other essential information were not related to HOTAIR (Table 1). Moreover, for the overall survival rate after radical surgery, patients with high HOTAIR expression group had a significantly poorer prognosis than those with low HOTAIR expression patients (*p* = 0.004; Fig. 1C). The univariate Cox regression analyses of overall survival showed that the lymph node invasion (*P* = 0.034), TNM stage (*P* = 0.023), postoperative recurrence (*P* = 0.011) and HOTAIR expression (*P* = 0.006) were good prognostic indicators. Furthermore, multivariate Cox regression analyses confirmed the expression of HOTAIR as an independent predictor for overall survival in patients with CCA (*P* = 0.039, Table 2).

**Table 1:** Association between HOTAIR expression and clinicopathological features of CCA.

Clinicopathological features	No. of patients(n)	HOTAIR expression		*p*-value
		High (n)	Low (n)	
Gender				
Male	33	20	13	0.544
Female	37	23	14	
Age				
<60	32	21	11	0.624
≥60	38	22	16	
Tumor site				
Intrahepatic	20	12	8	0.952
Perihilar	22	13	9	
Distal	28	18	10	
Tumor size				
<3	32	15	17	0.028
≥3	38	28	10	
Vascular invasion				
Positive	30	20	10	0.468
Negative	40	23	17	
Lymph node invasion				
Present	31	20	11	0.805
Absent	39	23	16	
TNM stage				
I–II	26	11	15	0.021
III–IV	44	32	12	
Differentiation grade				
Well/moderately	25	14	11	0.609
Poorly/undifferentiated	45	29	16	
Postoperative recurrence				
Present	53	37	16	0.020
Absent	17	6	11	
Serum CEA level				
>5 ng/ml	42	25	17	0.804
≤5 ng/ml	28	18	10	
Serum CA19-9 level				
>37 U/ml	46	27	19	0.609
≤37 U/ml	24	16	8	
HBV infection				
Positive	21	12	9	0.789
Negative	49	31	18

**Table 2:** Univariate and multivariate analysis of prognostic factors for overall survival in CCA patients.

Variables	Univariate analysis			Multivariate analysis		
	HR	95%CI	*P* value	HR	95%CI	*P* value
Overall Survival						
Gender (Mals vs. Female)	1.145	0.671–1.953	0.620			
Age (≥60 vs. <60)	1.001	0.587–1.709	0.996			
Tumor site (Extrahepatic vs. Intrahepatic)	1.540	0.847–2.800	0.157			
Tumor size (≥3 cm vs. <3 cm)	1.369	0.797–2.353	0.255			
Vascular invasion (Positive vs. Negative)	0.959	0.559–1.641	0.879			
Lymph node invasion (Present vs. Absent)	1.791	1.047–3.066	0.034	1.496	1.034–3.458	0.039
TNM stage (III–IV vs. I–II)	1.923	1.095–3.379	0.023	1.462	0.814–2.626	0.204
Differentiation grade (Well vs. Poorly)	1.160	0.904–2.876	0.106			
Postoperative recurrence (Present vs. Absent)	2.567	1.244–5.295	0.011	2.271	1.087–4.742	0.029
Serum CEA level (>5 ng/ml vs. ≤5 ng/ml)	1.387	0.798–2.413	0.246			
Serum CA19-9 level (>37 U/ml vs. ≤37 U/ml)	0.861	0.487–1.521	0.606			
HBV infection (Present vs. Absent)	1.439	0.825–2.510	0.200			
HOTAIR expression (High vs. Low)	2.251	1.260–4.022	0.006	1.891	1.034–3.458	0.039

### Overexpression of HOTAIR stimulates CCA cell proliferation

**Figure 2:**
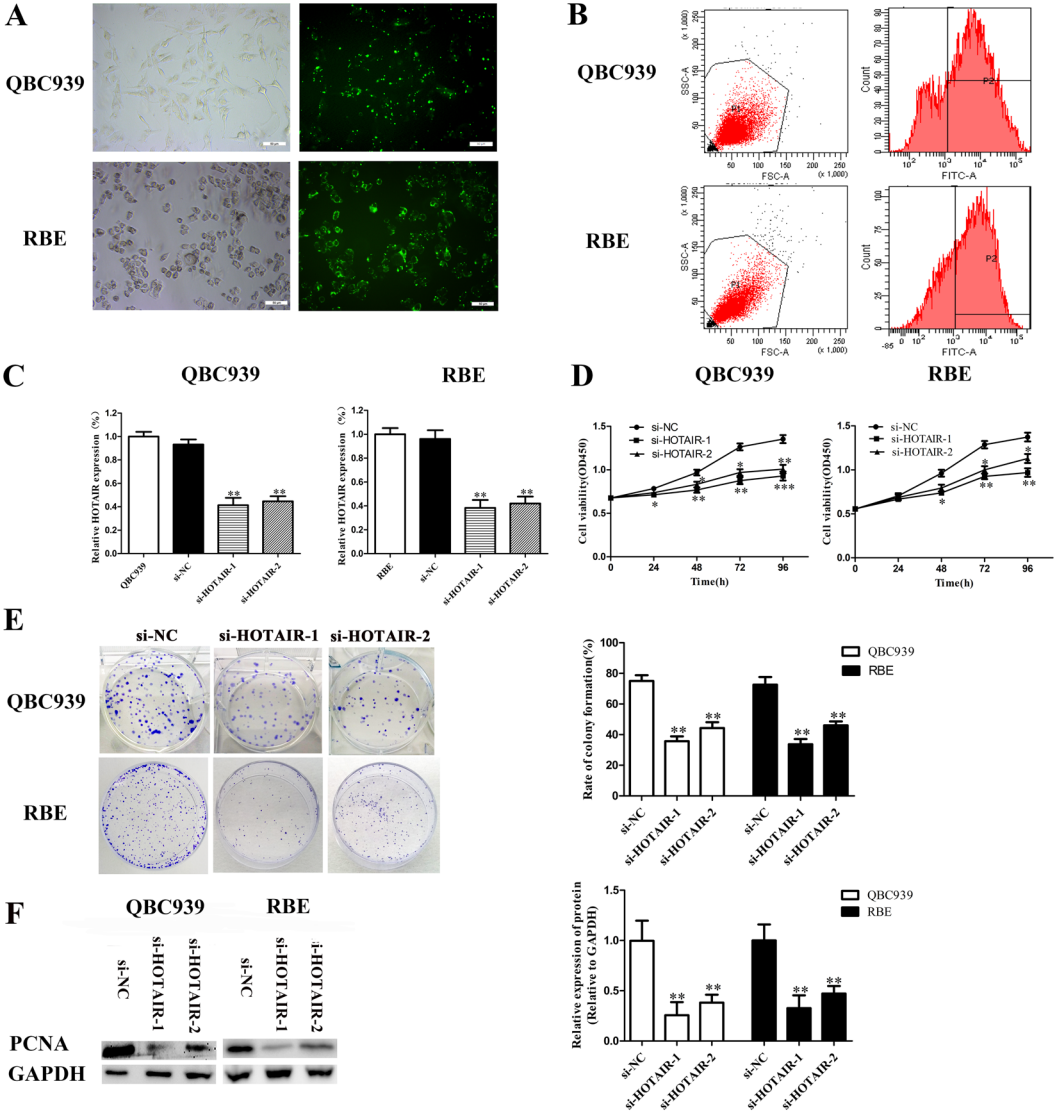
Results of HOTAIR after transfection and knockdown on cell proliferation and apoptosis. (**A**) Transfection efficiency detected by light microscope and fluorescence microscope after transfection; (**B**) Transfection efficiency of CCA cells (QBC939 and RBE) was detected by flow cytometry; (**C**) Relative expression of HOTAIR after transfected with si-RNAs by real-time PCR; (**D**) The proliferation of CCA cells (QBC939 and RBE) after transfection was measured by CCK-8 proliferation assay; (**E**) The colony formation capacity of CCA cells (QBC939 and RBE) after transfection was measured by clonogenic assay. (**F**) The protein level of PCNA in CCA cells (QBC939 and RBE) after transfection was measured by Western blot assay. ^*^p < 0.05, ^**^p < 0.01, ^***^p < 0.001.

In order to uncover the functional roles of HOTAIR in CCA *in vitro*, we designed two siRNAs with specifically targeting HOTAIR and an isotype control (si-NC) for transfection. We use the inverted fluorescence microscopy and flow cytometry to detect transfection efficiency. As for the two selected cell lines (RBE and QBC939), transfection efficiency reached 80–90% after transfection (Fig. 2A,B). Next, qRT-PCR was used to analyze the levels of HOTAIR expression in the two cell lines and as shown in Fig. 2C, the HOTAIR expression was significantly reduced after transfected with si-HOTAIRs. Cell Counting Kit-8 (CCK-8) was used to detect cell proliferation to assess the functional character of HOTAIR in CCA according to the instruction. The experimental data showed that the growth of QBC939 and RBE cells were both inhibited after knockdown of HOTAIR (Fig. 2D). And is similar with this should of is, colony formation assays showed that the clonogenic ability of two cell lines were significantly decreased after transfected with si-HOTAIR-1 or si-HOTAIR-2 (Fig. 2E). Western blot assays showed that the expression levels of proliferating cell nuclear antigen (PCNA) decreased significantly after knockdown of HOTAIR (Fig. 2F).

### The impact of si-HOTAIR in promoting CCA cell apoptosis

**Figure 3:**
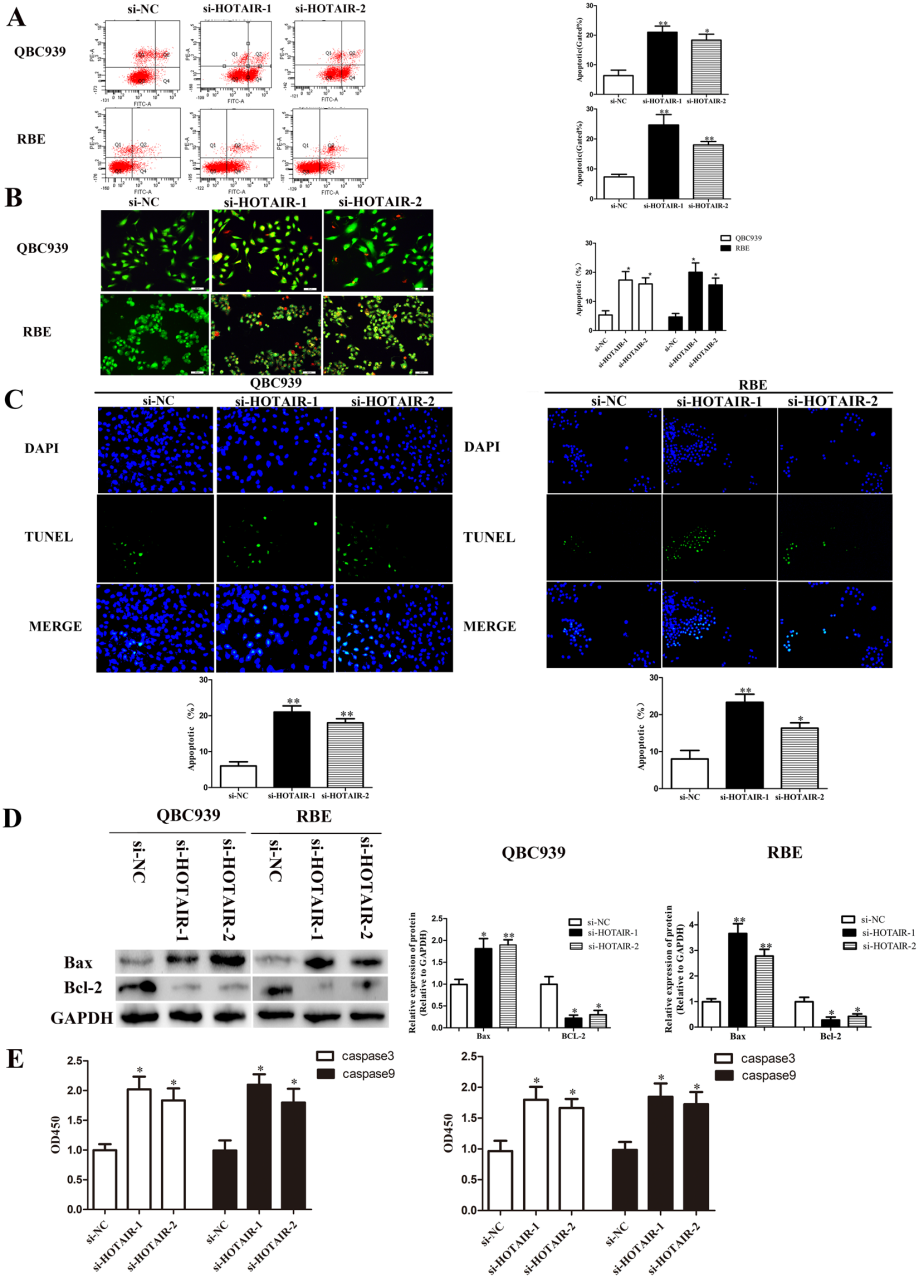
Knockdown of HOTAIR induced apoptosis in CCA cells. (**A**) The apoptosis of CCA cells (QBC939 and RBE) after transfection was evaluated by flow cytometry; (**B**) The apoptosis of CCA cells (QBC939 and RBE) after transfection was evaluated by AO/EB double fluorescence staining assay; (**C**) The apoptosis of CCA cells (QBC939 and RBE) after transfection was evaluated by TUNEL staining assay; (**D**) The protein levels of Bax and Bcl-2 in CCA cells (QBC939 and RBE) after transfection were detected by Western; (**E**) Relative expression of caspase-3 and caspase-9 in QBC939 and RBE cells after transfection were read by microplate reader. ^*^p < 0.05, ^**^p < 0.01.

In this study, we introduced acridine orange/ethidium bromide (AO/EB) double fluorescence staining, flow cytometry analysis, TdT-mediated dUTP Nick-End Labeling (TUNEL), caspase-3 and caspase-9 to explore the potential effect of HOTAIR on apoptosis of CCA cells. After transfected with si-HOTAIRs, the early and late apoptotic cells were both increased, nevertheless, most of cells were alive in the si-NC groups (Fig. 3A–C). Previous studies suggested that Bcl-2 family proteins could play an important role in cell growth and death. In our study, the expression of Bax protein were activated. However, the protein level of Bcl-2 were suppressed proved by Western blot analysis (Fig. 3D). Previous research had revealed that Bcl-2 family proteins act could regulators of cell life and death. But beyond that caspase-3, caspase-9, Bcl-2 and Bax are closely correlated with mitochondrial pathway mediated apoptosis. In our study, the expression of caspase-3 and caspase-9 were both activated after HOTAIR silenced (Fig. 3E).

### Knockdown of HOTAIR decreased the migration and invasion capacities of CCA cells

**Figure 4:**
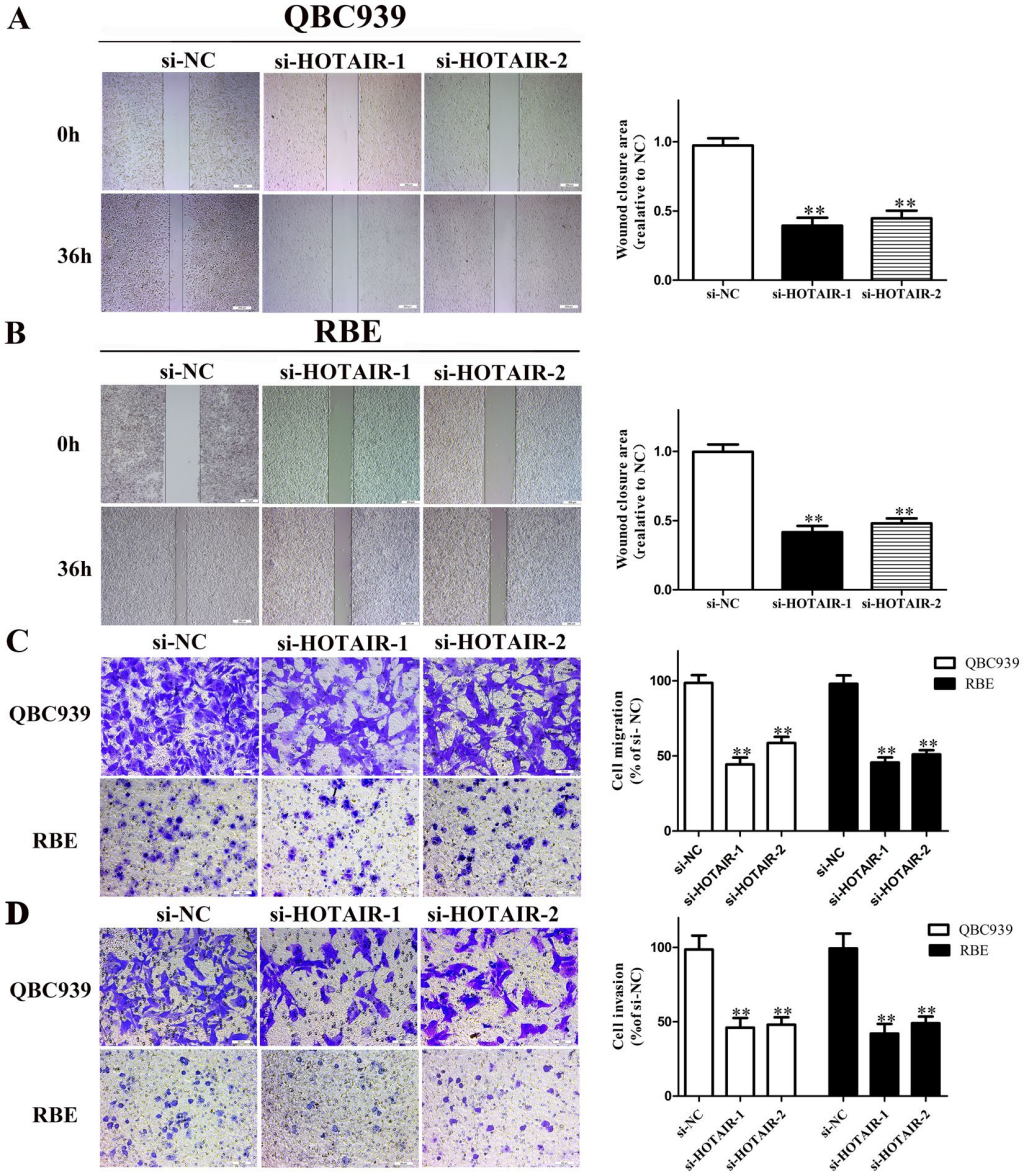
Knockdown of HOTAIR decreased cell migration and invasion potential in CCA cells. (**A**) The migration of CCA cells (QBC939 and RBE) after transfection was examined by wound healing assay; (**B**) The migration of CCA cells (QBC939 and RBE) after transfection was examined by Transwell migration assay; (**C**) The invasion of CCA cells (QBC939 and RBE) after transfection was examined by Transwell invasion assay. ^*^p < 0.05, ^**^p < 0.01.

In order to verify whether HOTAIR affects CCA cell migration and invasion, Transwell and wound healing assays were used after transfected with si-NC or si-HOTAIRs in QBC939 and RBE. Interestingly, as shown in Fig. 4A–D, after knockdown of HOTAIR, the migratory and invasion potentials were impeded compared with the si-NC groups. The results above indicated that down-regulation of HOTAIR distinctly suppressed RBE and QBC939 cell metastasis *in vitro*.

### Silenced HOTAIR affects EMT in CCA cells

**Figure 5:**
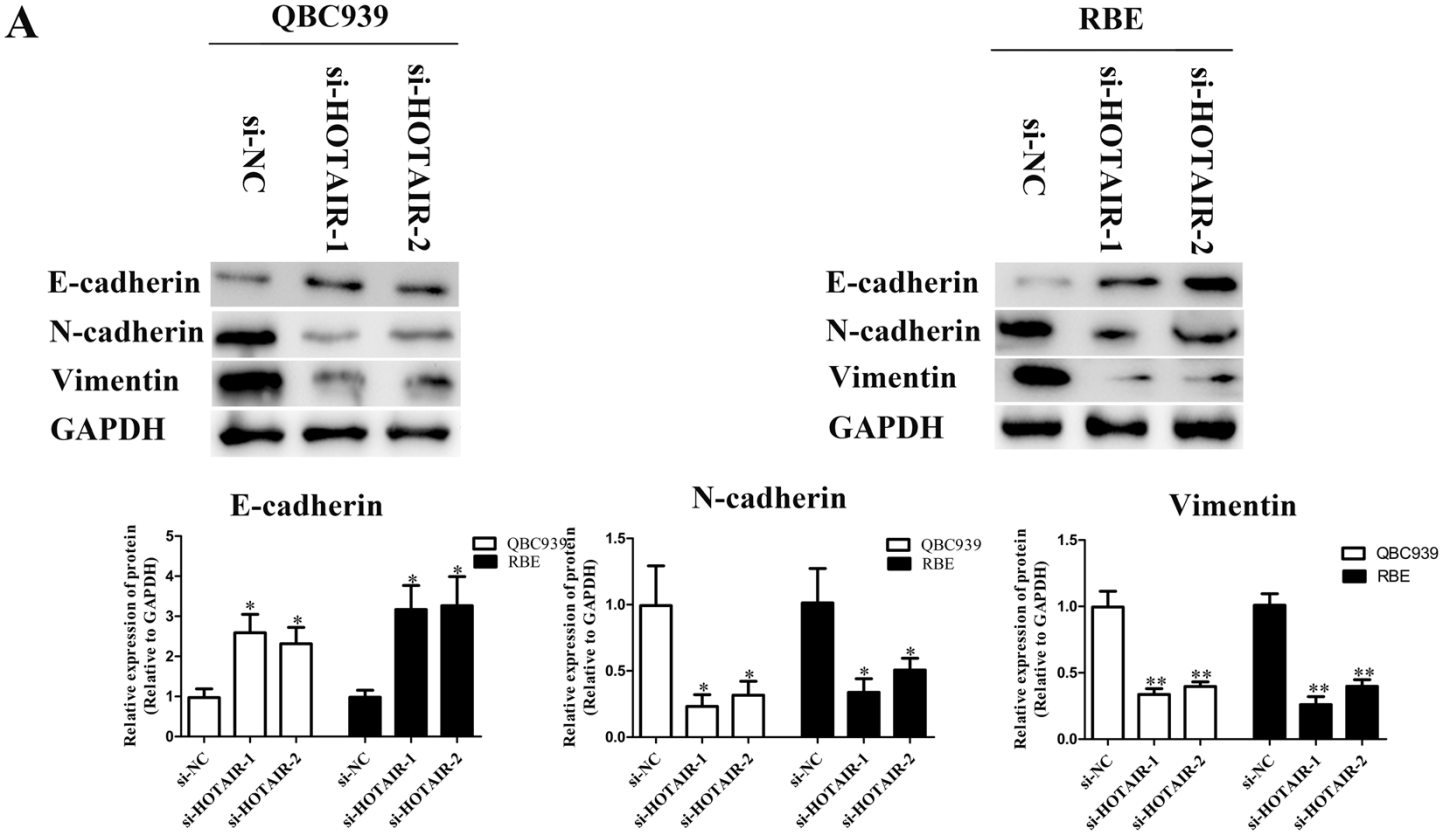
The expression of EMT after knockdown of HOTAIR compared with transfected si-NC. (**A**) The expression levels of EMT-related proteins in QBC939 and RBE cells after transfection were determined by Western blot assay. ^*^p < 0.05, ^**^p < 0.01.

EMT is an important mechanism related to cell metastasis, its mark including E-cadherin, N-cadherin and Vimentin are closely related to the epithelial cell invasion capacity. Therefore, Western blot were performed to investigate the relationship between dysregulation of HOTAIR and EMT. According to the Western blot results, the expression levels of N-cadherin and Vimentin were lower in QBC939 and RBE cells after HOTAIR was silenced, however, E-cadherin was significantly up-regulated (Fig. 5A).

### HOTAIR promoted CCA tumor formation and growth ***in vivo***

**Figure 6:**
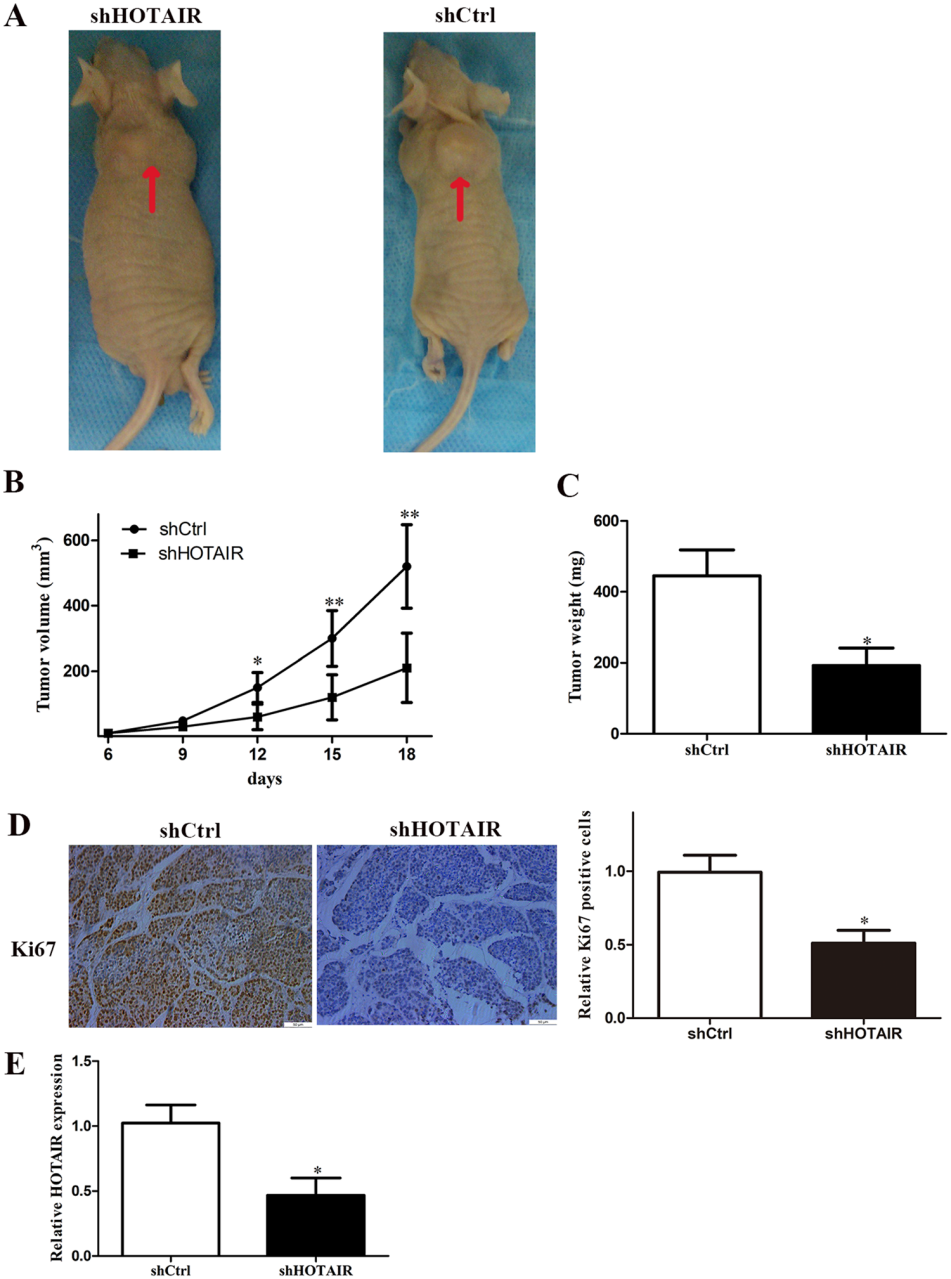
Effect of downregulated HOTAIR on tumorgenesis *in vivo*. (**A**) Tumors from nude mice after injection of QBC939 cells transfected with shHOTAIR or shCtrl. (**B**) Tumor volume was measured every 3 days after injection; (**C**) After 18 days of injection, the nude mice and tumor weights. (**D**) The Ki67 expression and positive cell numbers was determined by immunohistochemical staining. (**E**) qRT-PCR was performed to detect the average expression of HOTAIR. ^*^P < 0.05, ^**^P < 0.01.

To confirm whether the HOTAIR can effect tumorigenesis *in vivo*, shHOTAIR QBC939 cells and control cells were subcutaneously injected into BALB/c separately. As is shown in Fig. 6A,B, after inoculation 18 days, knockdown of HOTAIR significantly suppressed the growth of CCA xenografts. The average tumor weight in the shCtrl group was significantly higher than that in the shHOTAIR group (Fig. 6C). what’s more, immunohistochemical staining indicated a decreased proliferative index Ki67 expression in shHOTAIR group (Fig. 6D). The qRT-PCR results showed lower expression levels of the HOTAIR transcription in shHOTAIR group (Fig. 6E).

## Discussion

CCA is one of the most malignant tumors till now. Unfortunately, the incidence of CCA has increased significantly over the past few decades^1,21^. Though medical technology may improve the outcome of CCA patients, CCA still threatens people’s health due to its late presentation and lack of clinical treatments^22^. Given the loss of life, it is imperative to find new preventive strategies and reliable methods to treat the patients with CCA. Recently, the virtue of the lncRNAs has been well recognized, but the significant function also unwitnessed in relatively long term especially for variety of malignant tumors. Several lncRNAs have been confirmed to play an important role in various types of malignant tumors, including PANDAR, ZFAS1, HOTAIR, TUG1 and so on^23–26^. Emerging evidence indicated that in the process of tumor progression, lncRNA HOTAIR plays an imperative role^27,28^. However, its clinical significance and expression levels in CCA have not been studied yet.

HOTAIR, which originates from the HOXC cluster onchromosome, was the first lncRNA ever described to interact with polycomb proteins and suppress gene transcription. The high-expression of HOTAIR has been proved to play an imperative role in tumorigenesis and progression. Studies pointed out that lncRNA HOTAIR is overexpressed in breast cancer tissues compared with adjacent normal counterparts. Up-regulation of lncRNA HOTAIR was found in many other cancer tissues such as gallbladder cancer, pancreatic cancer, hepatocellular carcinoma, and nasopharyngeal carcinomas compared with their normal tissues^12,29–31^. These findings imply a potential role of HOTAIR in the development and progression of human cancers.

Recent studies have indicated that, in colon cancer, lncRNA loc285194 is a tumor-suppressive lncRNA through downregulating miRNA-211^32^. In gallbladder cancer, Ma *et al*. demonstrated that HOTAIR and miRNA-130a have a negative correlation. They found that the expression level of miRNA-130a is upregulated after HOTAIR silenced. In addition, the study found that knockdown of HOTAIR inhibited the invasiveness and proliferation of gallbladder cancer cells, miRNA-130a inhibitor reversed the effects of knockdown of HOTAIR exerted^12^. In hepatocellular carcinoma, Su *et al*. found that overexpression of HOTAIR is a FOXC1-activated promoter of malignancy^30^. Besides, in renal cell carcinoma, Dasgupta *et al*. found that MicroRNA-203 inhibits long noncoding RNA HOTAIR and regulates tumorigenesis through epithelial-to-mesenchymal transition pathway. Zhang *et al*. have proved that HOTAIR could enhances the Androgen-Receptor-Mediated transcriptional program and drives castration-resistant prostate cancer. Recent research by Ryunosuke *et al*. found that HOTAIR regulates polycomb-dependent Chromatin Modification and is associated with poor prognosis in colorectal cancers^20,33,34^. Consistent with the above research, our study confirmed that HOTAIR was overexpressed in CCA tissue samples compared with corresponding normal tissue samples. HOTAIR may be an oncogene and a negative prognostic factor in patients with CCA because high expression of HOTAIR was closely associated with the advanced TNM stage, larger tumor size, postoperative relapse and dismal 5-year overall survival.

CCK-8 and colony formation assays were used to determine the proliferation of CCA cells after suppressing HOTAIR expression. The results suggested that CCA cell multiplication capacities were affected by HOTAIR. PCNA is an essential factor in DNA replication, DNA repair, chromatin assembly and RNA transcription. Lots of researches have shown that amounts of PCNA were detected in normal proliferating cells as well as in transformed cells and tumors^35^. In our study, the results of Western blot experiments revealed that the expression of PCNA significantly decreased after knockdown of HOTAIR in CCA. Previous studies suggested that Bcl-2 family proteins could regulate cell growth and death. Bcl-2 and Bax could regulate tumor cell growth by regulating cell apoptosis rather than proliferation. In fact, it has been observed that overexpression of Bcl-2 or down-regulation of Bax can inhibit many tumor cell apoptosis, while down regulation of Bcl-2 or up-regulation of Bax can increase the apoptosis rate of tumor cells. What’s more, caspase-3 and caspase-9 play pivotal roles in the regulation of cell apoptosis among the 14 mammalian caspases identified to date. In addition, Receptor mediated pathway and mitochondrial pathway are related to the regulation of Bax, Bcl-2, caspase-3 and caspase-9. Moreover, in our study Bax was upregulated after knockdown of HOTAIR, on the other hand, Bcl-2 with low expression levels, this is probably an indirect argument that HOTAIR promotes tumor development and progression in CCA^36–40^. Other experiments including flow cytometric analysis and AO/EB staining assays were also determined that as the HOTAIR expression decreased, the rate of apoptosis increased. Transwell and scratch wound assays showed that knockdown of HOTAIR with siRNAs significantly reduced the migration and invasion of QBC939 and RBE cells. EMT has been proved to play an important role in tumor invasiveness, metastasis and prognosis^41,42^. Recent researches indicated that N-cadherin, Vimentin and Fibronectin are upregulated, however, E-cadherin and Claudins are down-regulated. It has been reported that it is an essential step in the transformation between E-cadherin and N-cadherin to manipulate cell metastasis in CCA^43^. Knockdown of HOTAIR could reverse EMT progress and then impair migratory and invasive potential of QBC939 and RBE cells proved by Western blot analysis. However, there are still some limitations to come to an incontrovertible conclusion in our research. A larger cohort of patients should be recruited to further validate the clinical value of HOTAIR in CCA. The mechanisms underlying the regulatory actions of HOTAIR should be further investigated.

In conclusion, these studies indicated that HOTAIR was remarkedly upregulated in CCA tissues and cell lines compared with normal tissues and HIBEC. Our findings identified an oncogenetic role of HOTAIR in the tumorigenesis and development of CCA both *in vitro* and *in vivo*. Upregulated HOTAIR may be a negative prognostic factor for CCA patients. Besides, HOTAIR may become a novel therapeutic target for the treatment of CCA.

## Materials and Methods

### Patients and Tissue collection

70 paired tissue and corresponding adjacent non-tumorous tissues were obtained from patients who underwent surgery at the Second Affiliated Hospital of Harbin Medical University between 2010 and 2013 and no patients underwent radiotherapy and chemotherapy treatment. The study was approved by the Ethics Review Committees of Harbin Medical University, and written informed consents were obtained from all patients. We confirm that all methods were performed in accordance with the relevant guidelines and regulations.

### Cell lines and culture

Two CCA cell lines (HCCC-9810 and RBE) were commercially obtained from the Cell Bank of Chinese Academy of Sciences (Shanghai, China). Human intrahepatic biliary epithelial cells (HIBEC), and another CCA cell lines including QBC939, Huh-28, HuCCT1 and CCLP-1 were preserved in our laboratory. Cells were cultured in RPMI 1640 or dulbecco’s modified eagle medium (DMEM) medium supplemented with 10% fetal bovine serum (FBS), 100 U/ml penicillin and 100 mg/ml streptomycin (Invitrogen, Carlsbad, CA, USA) in humidified air with 5% CO_2_ at 37 °C.

### RNA extraction and quantitative real-time PCR

Trizol reagent (Thermo Fisher Scientific, Waltham, MA, USA) was used to extract total RNA from tissues or cultured cells. qRT-PCR assays were performed by using FastStart Universal SYBR Green Master (Roche, Germany) in a BIO-RAD C1000 Thermal Cycler, and total RNA was subjected to cDNA by Transcriptor First Strand cDNA. GAPDH was selected as the negative control. The primers used for GAPDH and HOTAIR were as follows: HOTAIR Forward, 5′-GGGAGCCAAAAGGGTCAT-3′ and Reverse, 5′-GAGTCCTTCCACGATACCAA-3′; GAPDH Forward, 5′-GGGAGCCAAAAGGGTCAT -3′ and Reverse, 5′-GAGTCCTTCCACGATACCAA -3′.

### siRNAs and transfection

RBE and QBC939 were selected for the knockdown study, on the basis of the expression of HOTAIR in CCA cell lines. We cultured cells in serum-free medium and allowed them to grow to half confluence prior to transfected with si-HOTAIR using Lipofectamine 3000 transfection reagent (Thermo Fisher Scientific) for 48 h. The target sequences for si-HOTAIR are as follows: si-HOTAIR-1 sense 5′-UUCUAAAUCCGUUCCAUUCCACUGCGA-3′; antisense 5′-GCAGUGGAAUGGAACGGAUUUAGAA-3′; si-HOTAIR-2

sense 5′-AGCGAACCACGCAGAGAAAUGCAGG-3′; antisense

5′-CCUGCAUUUCUCUGCGUGGUUCGCUUU-3′. The sequence for negative control FAM is: sense 5′-UCUCCGAACGUGUCACGUTT-3′; antisense 5′-GUGACACGUUCGGAGAATT-3′.

### Proliferation assays

For CCK-8 analysis, in 96-well plates, seeding 1500 transfected cells/well after QBC939 and RBE cells were transfected with si-HOTAIR or si-NC. Using CCK-8 (Dojindo, Tokyo, Japan) to detect viability at following time (0, 24, 48, 72 and 96 h), added 10 uL of CCK-8 into the corresponding wells. A microplate reader (Tecan, Männedorf, Switzerland) was used to analyze the absorbance at 450 nm after incubated at 37 °C for 2 h. For the clonogenic assay, QBC939 and RBE cells were trypsinized into a single-cell suspension and plated in a 3.5-cm dish at a total of 500 cells per well. The cells were maintained in an incubator for approximately 2 weeks until there were visible colonies.

### Flow cytometry for cell apoptosis

Collecting QBC939 and RBE cells after transfected with si-NC or si-HOTAIR and washed twice with cold PBS. Binding buffer was used to re-suspend the cells. After staining with 5 μL FITC-Annexin V and 5 μl Propidium iodide (PI) using FITC Annexin V Apoptosis Detection Kit (BD, Biosciences, USA), the stained cells were measured by flow cytometry (FACScan; BD Biosciences, USA).

### Acridine orange/ethidium bromide (AO/EB) double fluorescence staining

The exponential growth phase cells were cultured in an incubator of 5% CO_2_ at 37 °C and transfected with si-HOTAIR or si-NC, and then, stained with prepared AO/EB mixing solution for 5 min (Solarbio, Beijing, China). Because of different abilities to penetrate the cell membrane, AO/EB could tell live cells from apoptotic cells. Apoptotic cells DNA were dyed red or orange while the normal cells with green fluorescence. At last, the fluorescence microscope (Leica, Germany) was used to take photographs and determined the apoptotic cells.

### TdT-mediated dUTP Nick-End Labeling (TUNEL) assay

To explore the apoptotic in QBC939 and RBE cells, apoptosis was examined using One Step TUNEL Apoptosis Assay Kit (Beyotime, Beijing, China). Transfected cells were washed by PBS after fixed with paraformaldehyde for 30 min. Then, the cells were incubated by Immunostaining Permeabilization Buffer with Triton X-100 (Beyotime, Beijing, China). Afterwards, QBC939 and RBE cells transfected with si-NC or si-HOTAIR were incubated at 37 °C for 60 min after treated with 45 μl fluorescent labeled reagent and 5 μl terminal deoxynucleotidyl transferase (TdT). A fluorescent microscope (Leica, Germany) was used to detect the apoptotic cells.

### Relative caspase activity determination

After post-transfection 48 h by using Caspase-3 Activity Kit and Caspase-9 Activity Kit (Solarbio, Beijing, China). The relative of caspase-3 and caspase-9 activity were detected. Add cell proteins into 96-well plates with reaction buffer and substrate and then maintained at 37 °C for 4 h in the dark before quantified at a wavelength of 405 nm by a microplate reader (Tecan, Männedorf, Switzerland).

### Scratch wound assay

RBE and QBC939 cells were transfected by si-HOTAIR or si-NC and separately planted in 3.5 cm dishes until reaching about 80% confluence. Using phosphate buffer solution (PBS) to clean up cellular debris after mark out a straight wounds by a sterile 200-μL pipette tip. Migration distance were taken photographs after 0 h and 36 h.

### Migration and invasion assays

Using transwell chambers with 8-μm polycarbonate nucleopore filters (Corning, NY, USA) to determine the alteration of migration and invasion capacities. 5 × 10^4^ transfected cells were seeded in 200 ul serum-free medium loaded in the top section of a 24-well transwell. The bottom was loaded 600 ul complete medium. After the transwell chambers were incubated for 24 h, crystal violet was used to stain the cells on the lower surface. For invasion, first, put the Transwell chamber unit in 37 °C for 4 h after coated with 40 μl Matrigel (BD Biosciences, San Jose, CA, USA) in the top of Transwell chamber unit to reconstruct basement membrane. And then according to the methods of the migration assay.

### Western blot analysis

RIPA buffer with protease inhibitors (Beyotime, Beijing, China) were used to lysed QBC939 and RBE cells at 48 h post-transfection. Sodium dodecyl sulfate polyacrylamide gel (SDS-PAGE) vertical electrophoresis were used to fractionated the extracted proteins. The proteins were transfered to a 0.45 μm polyvinylidene fluoride (PVDF) membrane (GE Healthcare, Piscataway, NJ, USA). Afterwards, 5% defatted milk diluted in Tris-buffered saline containing 0.05% Tween-20. Primary antibodies include GAPDH, E-cadherin, N-cadherin and Vimentin were used to incubate the membranes overnight. Then, the membranes were incubated in secondary antibody (Cell Signaling Technology, Danvers, USA) for 2 h before using eyoECL Plus Kit (Beyotime, Beijing, China) to visualize the blots.

### Tumor xenografts study

All animal studies were performed under the supervision and guidelines of the Animal Care and Use Committee of Harbin Medical University. We confirm that the experimental protocol was approved by Animal Care and Use Committee of Harbin Medical University. 3 × 10^6^ QBC939 cells were transfected with shHOTAIR and subcutaneously injected into either side of the flank area of 6-week-old female BALB/c nude mice (n = 3 per group). Tumor size was measured every 3 days and tumor volumes were calculated using the equation: V = 0.5 × D × d^2^ (V, volume; D, longitudinal diameter; d, latitudinal diameter). The nude mice were euthanized after injected 18 days, and the tumors weight were measured.

### Immunohistochemistry study

Immunohistochemistry study was carried out on the paraffin-embedded tumor tissues from nude mice. The avidin-biotin-peroxidase method were used to explore the location and relative expression level of Ki67 protein. Image-Pro Plus 6.0 software was used to quantify the number of positive cells in each image. The results were expressed as the average percentage of positive cells per image. The Ki67 primary antibody (Abcam, Cambridge, MA, USA) was used at a dilution of 1:200.

### Statistical analysis

All the experiments were totally repeated for 3 times. The results are reported as the means ± standard deviation (SD) based on at least three replicates. The methods of calculating power of study were performed by using GraphPad Prism 5.01 software (GraphPad Software, Inc., La Jolla, CA, USA) and SPSS 20.0 statistical software package (IBM, Armonk, NY, USA). A paired and two-tailed Student’s t-tests were used to evaluate the significant differences between groups. Fisher’s exact test was used to analyze the link between HOTAIR expression and clinicopathological features. Survival curves were drawn by Kaplan-Meier analyses and tested using log-rank tests. Survival data were analyzed by using univariate and multivariate Cox proportional hazards modeling. The data were considered significant only when p-values less than 0.05.
